# Behavioral variation according to feeding organ diversification in glossiphoniid leeches (Phylum: Annelida)

**DOI:** 10.1038/s41598-021-90421-1

**Published:** 2021-05-25

**Authors:** Hee-Jin Kwak, Jung-Hyeuk Kim, Joo-Young Kim, Donggu Jeon, Doo-Hyung Lee, Shinja Yoo, Jung Kim, Seong-il Eyun, Soon Cheol Park, Sung-Jin Cho

**Affiliations:** 1 Department of Biological Sciences and Biotechnology, College of Natural Sciences, Chungbuk National University, Cheongju, Chungbuk 28644 Republic of Korea; 2 Wildlife Disease Response Team, National Institute of Wildlife Disease Control and Prevention, Incheon, 22689 Republic of Korea; 3 Department of Life Sciences, Gachon University, Gyeonggi-do, 13120 Republic of Korea; 4 Department of Life Science, Chung-Ang University, Seoul, 06974 Korea; 5 Department of Molecular and Cell Biology, University of California, 385 LSA, Berkeley, CA 94720-3200 USA; 6 Department of Molecular and Cell Biology, University of California, 539 LSA, Berkeley, CA 94720-3200 USA

**Keywords:** Developmental biology, Evolution

## Abstract

Adaptive radiation is a phenomenon in which various organs are diversified morphologically or functionally as animals adapt to environmental inputs. Leeches exhibit a variety of ingestion behaviors and morphologically diverse ingestion organs. In this study, we investigated the correlation between behavioral pattern and feeding organ structure of leech species. Among them, we found that *Alboglossiphonia* sp. swallows prey whole using its proboscis, whereas other leeches exhibit typical fluid-sucking behavior. To address whether the different feeding behaviors are intrinsic, we investigated the behavioral patterns and muscle arrangements in the earlier developmental stage of glossiphoniid leeches. Juvenile Glossiphoniidae including the *Alboglossiphonia* sp. exhibit the fluid ingestion behavior and have the proboscis with the compartmentalized muscle layers. This study provides the characteristics of leeches with specific ingestion behaviors, and a comparison of structural differences that serves as the first evidence of the proboscis diversification.

## Introduction

Diverse animals have evolved a great variety of ways to obtain the energy needed for their survival and reproduction. Some invertebrates and vertebrates use potent jaws to swallow the entire prey (macrophagous)^1–5^, while others use organs such as proboscises or stylets to penetrate the body wall of the prey and suck out fluid (fluid ingestion)^6–11^. Leeches (Phylum Annelida), are within the class Clitellata, superorder Euhirndinea. The group of Clitellata consists of Euhirudinea, Acanthobdellida, Branchiobdellida, and Oligochaeta, all of which are hermaphroditic and deposit cocoons through a special organ called clitellum^12^. Leeches are either carnivorous or ectoparasitic and feed on a wide range of prey. Accordingly, they exhibit a wide variety of ingestion behaviors, morphologically diverse mouthparts, and specialized guts. Previous studies have partly classified leeches based on the differences in their feeding structures (e.g., Rhynchobdellida, and Arhynchobdellida)^13,14^, and these groupings have been supported by modern molecular phylogenies. Representative classification of Hirudinea is as follows: Hirudiniformes comprise jawed leeches, which use jaws with teeth to injure the host’s body wall and ingest body fluids; Rhynchobdellida consists of jawless leeches, which use the proboscis to penetrate the host’s body wall and ingest body fluids; and Erpobdelliformes are jawless leeches that swallow foods^13,15–17^. The food ingestion behaviors of leeches have been identified previously; however, studies investigating the differences in internal organ structure according to behavioral patterns are limited to selected species.

Among the fluid ingestion leeches, the glossiphoniid leeches display diverse trophic levels in the ecosystem^6,13,17–21^. Their food consumption behavior shows a consistent and stereotyped pattern involving a structure called proboscis which is used to penetrate a host’s body wall and ingest body fluids. In the well-studied leech model *Helobdella austinensis*, a clitellate annelid, proteoblast DM’’ (labeled as the 4d cell in Spirallian nomenclature) contributes to development of an unsegmented prostomium which develops into a proboscis during organogenesis^22,23^. The proboscis is comprised of longitudinal, radial and circular muscles, which it uses to extend forward and backward to penetrate the body wall of the host and ingest body fluids^13^. In the Lophotrochozoans, the striated muscle specific gene *st-mhc* and troponin complex are known to be involved in the development of foregut muscles as well as somatic muscles^24,25^. These studies hint at the existence of foregut specific factors, which further implies the potential for visualization of these factors within the foregut muscle structure. Additionally, some studies have suggested that these foregut-specific muscle complexes may be regulated by innervation^24,26^. Therefore, we sought to characterized the muscle arrangement in the foregut region of leeches using in situ hybridization and immunohistochemical analyses.

Glossiphoniid leeches are known for their typical feeding behaviors that rely on their whip-shaped proboscises^12,13^. *Theromyzon tessulatum*, a parasitic leech that feeds from the nasal passages of aquatic birds, and *Placobdella costata*, an ectoparasitic leech of freshwater turtles, have proboscises that consist of outer longitudinal muscle, circular muscle that circumscribe the lumen, and radial muscle fibers^13^. However, the relationship between internal structure and ingestion behavior is not well understood. Here, we investigate the internal structure of proboscises in the family Glossiphoniidae. Interestingly, despite *Alboglossiphonia* sp. representing distinct morphological and molecular phylogenetic features of genus *Alboglossiphonia*, which belongs to the family Glossiphoniidae, it shows macrophagous feeding behavior by surrounding and swallowing a prey whole.

In this study, we demonstrate the characteristics of leeches with different types of ingestion behaviors, and investigate molecular level structural differences, providing the first evidence of proboscis diversification in the family Glossiphoniidae.

## Results and discussion

### External morphological features and phylogenetic status of leeches

**Figure 1:**
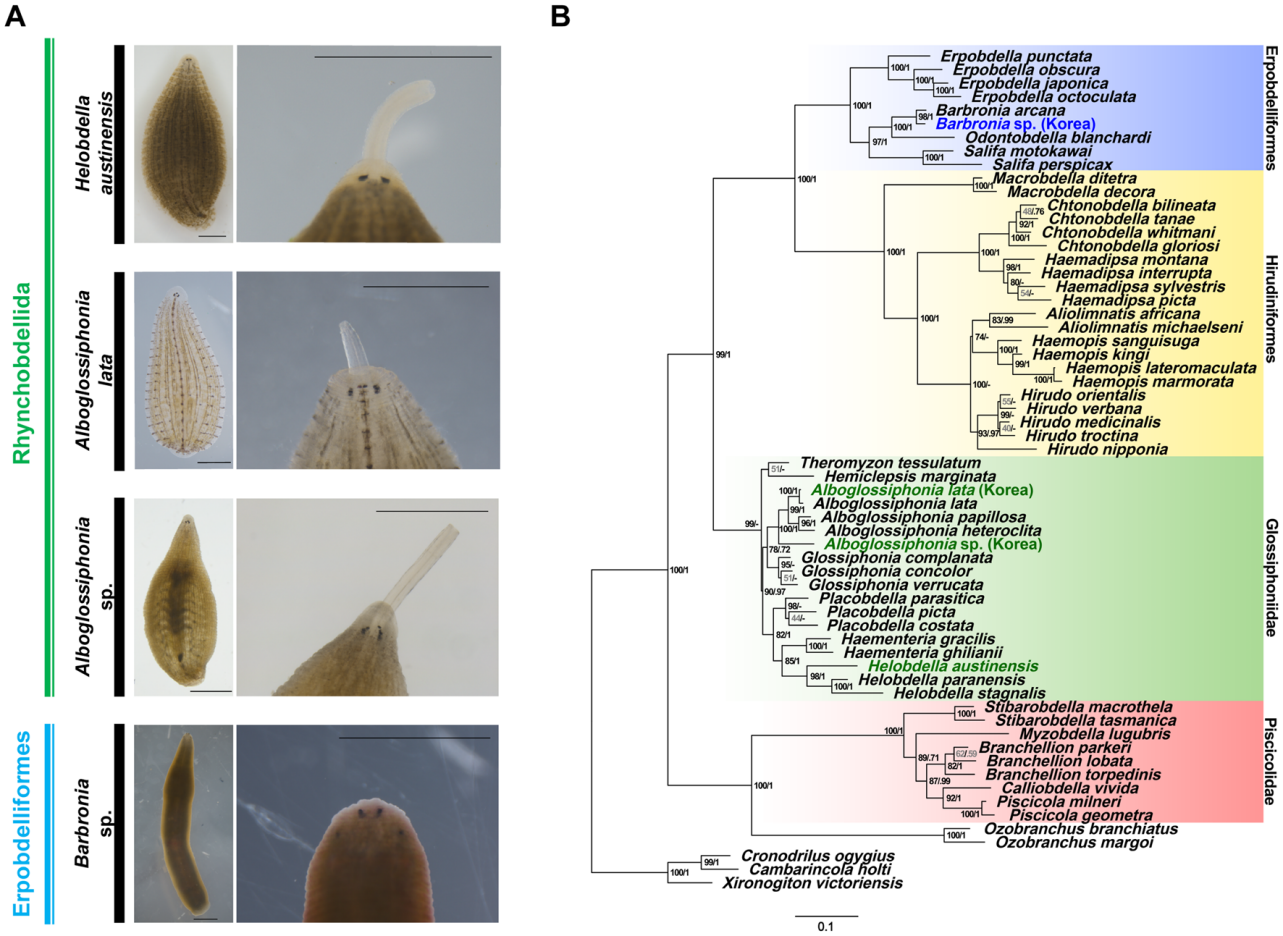
External morphological features and phylogenetic status of leeches. (**A**) Three glossiphoniid leeches (*Helobdella austinensis*, *Alboglossiphonia lata*, and *Alboglossiphonia* sp.) have a retractable proboscis that is characteristic of Rhynchobdellida in contrast to erpobdelliformes specimen *Barbronia* sp. (Scale bars 2 mm). (**B**) Maximum likelihood (ML) phylogeny based upon the concatenated sequences of CO1 and 18S rRNA including three branchiobdellid taxa (*Xironogiton victoriensis*, *Cronodrilus ogygius* and *Cambarincola holti*) as outgroups. The ML tree was estimated under the GTR + I + G (4 gamma categories) model with 3000 bootstrap replicates. The numbers near branching points indicate the transfer bootstrap expectation (TBE) supports (BS, in percentage) and Bayesian Posterior Probabilities (PP, in probability) and are presented as “BS/PP”. Dashes (−) after BS indicate PP that has not been applicable for the ML tree mainly due to the topological discrepancies between ML and Bayesian Inference (BI) trees. For BI topology, see Supplementary Fig. S1B.

The present molecular phylogenetic analysis shows a clear separation of the four main clades of leeches, Erpobdelliformes, Hirudiniformes, Glossiphoniidae, and Piscicolidae, with strong branch-support values (Fig. 1B). This result is generally congruent with the conventional classification based on their morphological characteristics (Fig. 1 and Supplementary Fig. S1)^27^. All species of *Alboglossiphonia* with a proboscis are placed within Glossiphoniidae, a group made up of jawless leeches (fluid-ingestion leech group). Within this group, *Alboglossiphonia* sp. has an extraordinary feeding behavior, swallowing prey whole using its proboscis (Supplementary Movie 1). Macrophagy is a representative characteristic of Erpobdelliformes^15^. The phylogenetic relation between the Erpobdelliformes group and *Alboglossiphonia* sp. is, however, unsupportable with current molecular result due to their separation at an earlier node. The genus *Alboglossiphonia* forms a monophyletic group within Glossiphoniidae with relatively high branch supporting values (BS = 100%, and PP = 1.00), but the origin of the ingestion behavior, which differs from that of the close congener *Alboglossiphonia lata,* remains unclear. In other words, it is difficult to explain the behavioral features based on external and molecular phylogenetic characteristics. From another perspective, these issues raise questions about the types of unique changes that may occur within the same family. Also, the findings suggest that the ingestion characteristics of *Alboglossiphonia* sp. arise from changes in the internal structure of esophagus.

### Comparative ingestion behavior of leeches with different food sources

**Figure 2:**
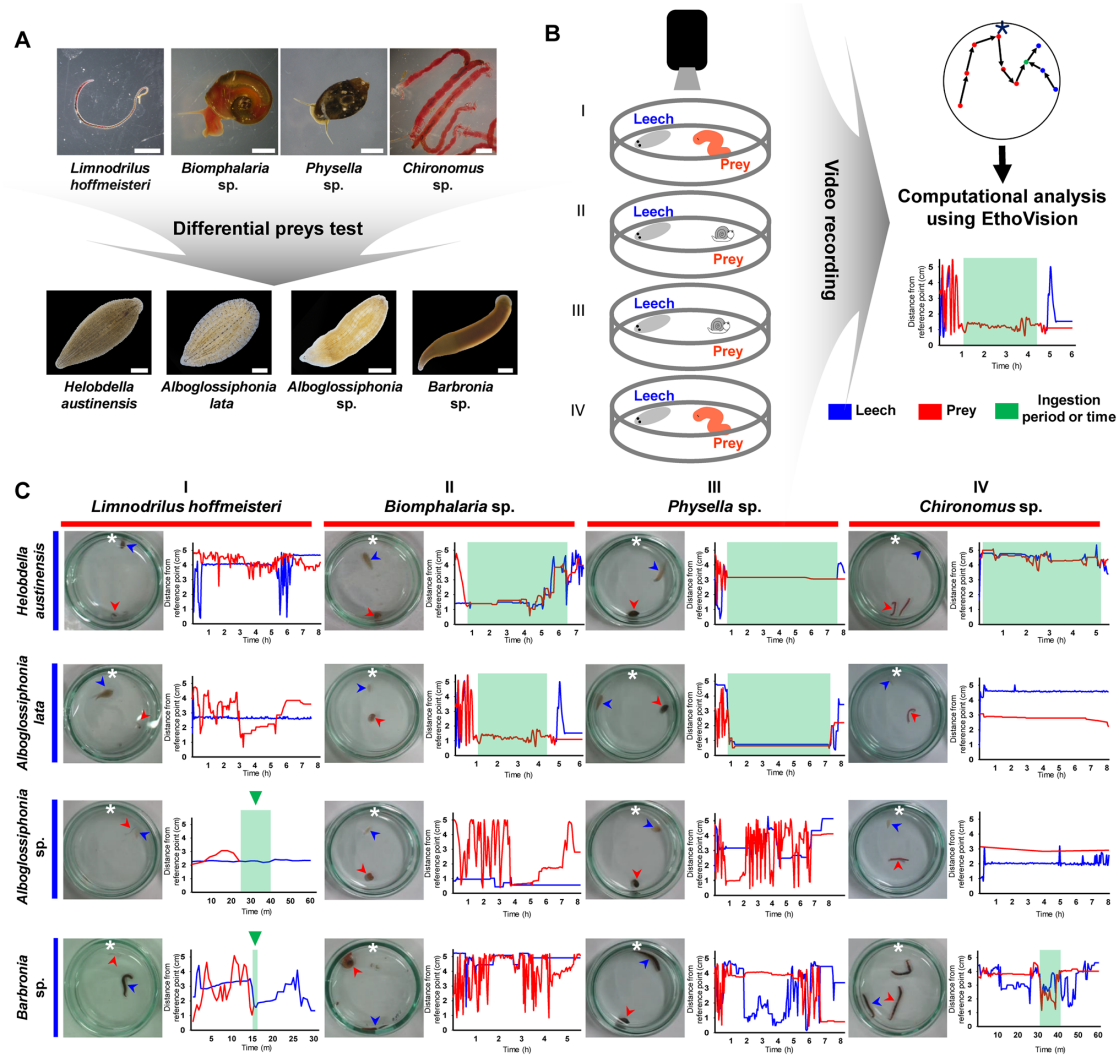
Comparative ingestion behavior of leeches with different food sources. (**A**) Representative leech species and prey types. See also Supplementary Movies 1–4 for detailed ingestion behavior. Scale bars 2 mm. (**B**) Schematic procedure of different ingestion tests depending on prey type. The recorded locomotion of a leech and its prey was analyzed using EthoVision, a target tracking program. The relative distance from leech (shown in blue) or prey (shown in red) to the reference point (asterisk) was measured. Ingestion period is indicated by green box. (**C**) Representative behaviors of leeches in the presence of specific prey. Each graph represents the distance between the leech (blue arrowhead) and the food (red arrowhead) from the reference point (white asterisk). When a fluid sucking leech adhered to food, its position was consistent (green box) for a period of time. After ingestion of food, the remaining prey that cannot be swallowed persisted. In the case of macrophagous leeches, only locations of the leech remained detectable (green box with green arrowhead) after ingestion of whole prey targets by the leech. Only *Limnodrilus hoffmeisteri* was fully ingestible by macrophagy.

Leech species exhibit either overlapping or unique trophic niches and thus display diverse feeding behaviors based on the target food sources^6,13,20,28^. In order to observe the exact feeding behavior patterns of different leech species, behavioral experiments involving various feeding conditions are required. To date, most reported analyses have focused on quantitative evaluation or positive reaction based on serological tests^13,28–30^. Therefore, we first investigated the food preferences and differences in feeding behaviors of leeches using various prey that had been reported on previously^3,6,13,19^. We tested the following prey species in the present study to determine the specific ingestion behavior of leeches: *Limnodrilus hoffmeisteri*, a vermiform freshwater oligochaete that can be swallowed whole; *Biomphalaria* sp. and *Physella* sp., freshwater snails that cannot be swallowed by leeches; and *Chironomus* sp., an insect larva that is unswallowable due to its large size and/or presence of cuticle (Fig. 2). *H. austinensis* ingests the body fluids of bloodworms and snails by inserting its proboscis into the host’s body wall thereby sucking out the body fluids as reported previously^6^. A second glossiphoniid species, *A. lata*, exhibits a feeding behavior pattern very similar to *H. austinensis*. It attacks only snails, and does so by inserting its anterior end into the snail’s shell and sucking the body fluids through its inserted proboscis (Fig. 2C, Supplementary Movies 2 and 3). Unlike other glossiphoniid leeches, *Alboglossiphonia* sp. intakes only freshwater earthworms through a macrophagous feeding behavior in which it wraps around the prey and eats it whole, similar to *Barbronia* sp. (Fig. 2C, Supplementary Movies 1 and 4). Through food preferences, we identified the unique trophic niches occupied by the sympatric leeches *Alboglossiphonia* sp. and *A. lata* under non-competitive conditions. We assumed that, despite living in the same habitat, the trophic niche partitioning would allow these leeches to coexistence without competition^31–33^. Also, unique ingestion behaviors support the contention that the structure of feeding organ in *Alboglossiphonia* sp. is different from that of the other glossiphoniid leeches and resemble that of macrophagous leeches.

### Comparative structural morphology of leech feeding organs

Species in different ecosystems have evolved to suit different habitats by undergoing changes in their external and internal structure, and manifesting behavioral variations that allow them to survive various external pressures^34–36^. These changes can often result in behavioral convergence, where different species exhibit similar behaviors due to a structure or behavior being advantageous in both environments (e.g., Rodent turbinate; Arachnid web architectures)^37,38^. Among various environmental factors, the specific behavioral convergence about food that is essential for survival manifests in various aspects (e.g., Ultrasonic predator whale and bat)^39,40^. These behavioral convergences cannot be explained phylogenetically, suggesting that behavioral convergence is the result of evolutionary convergence depending on the choice of similar food sources via speciation. Thus, the similar ingestion behaviors of *Alboglossiphonia* sp. and *Barbronia* sp., suggest possible differences in the proboscis of *Alboglossiphonia* sp. from others in the family Glossiphoniidae. In the present study, the structure of the ingestion tube was elucidated via histological and molecular analyses in order to identify the structural similarities and differences between these leeches’ feeding structures.

**Figure 3:**
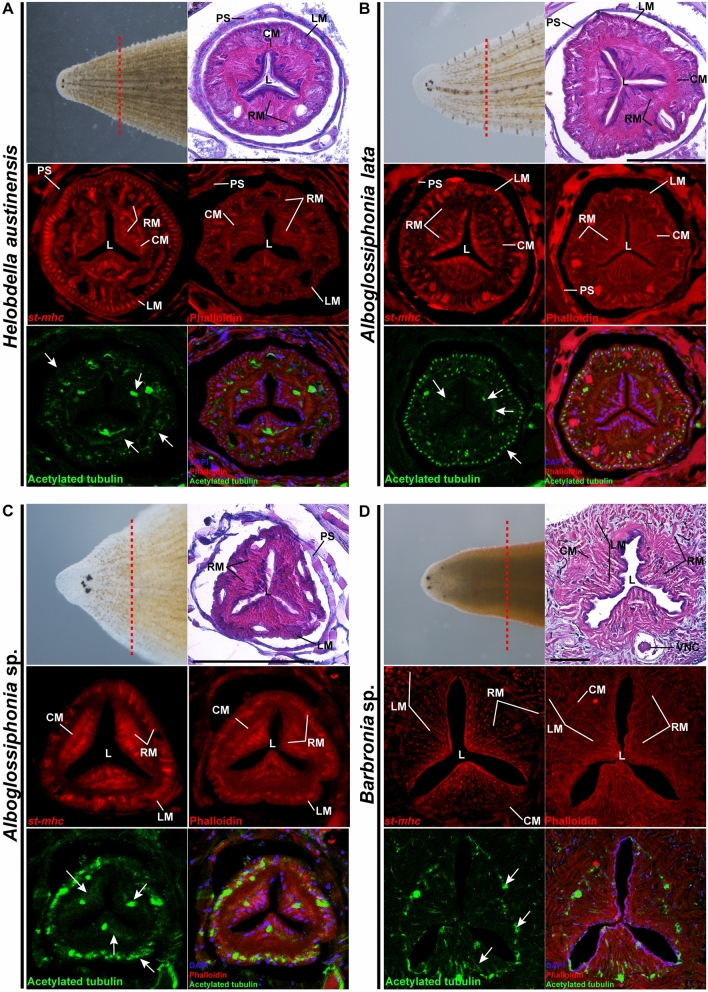
Comparative structural morphology of leech feeding organs. (**A**–**D**) Histological analyses of cross sections of the leech proboscis. The upper left corner of each set of cross-sectional images shows a dorsal-anterior view of the leech species and the sectional region (red dashed lines). Longitudinal, circular and radial muscle structures are labeled with H&E staining (top right rows) and *st-mhc* ortholog expression patterns (middle left rows). Fluorescent labeling of neuronal (white arrow) and muscular structures by anti-acetylated tubulin and phalloidin, respectively. DAPI staining was performed to visualize the entire morphology of proboscis by labeling nuclei. *H. austinensis* and *A. lata* exhibit clear compartmentalization of the innervated muscle layers (**A**,**B**). *Alboglossiphonia* sp. has a proboscis, but does not have a clear distribution of muscle layers in the histological analysis; only the outer longitudinal muscle and the partial circular muscle layers are identifiable (**C**). *Barbronia* sp., which has esophagus, shows an extended lumen with a circular muscle, and the inner cavity is composed of a complex of radial and longitudinal muscles (**D**). Red dotted lines represent the top view of the cross section. *CM* circular muscle; *L* lumen; *LM* longitudinal muscle; *PC* proboscis cavity; *RM* radial muscle; *PS* proboscis sheath; *VNC* ventral nerve cord. See also Supplementary Fig. S2B for structural details of histological analyses. Scale bars 150 μm.

The proboscis of glossiphoniid leeches is a muscularized tube-like organ specialized for penetration into prey and ingestion of the prey’s blood or other body fluids and soft tissues. Developmentally, the proboscis arises primarily from mesodermal precursor cells known as M teloblasts. Structurally, it is characterized by a sharply defined complement of longitudinal, radial, and circumferential muscles^22^. Longitudinal muscles form the outer edge of the proboscis and radial muscles span the thick wall of the proboscis from just within the longitudinal muscles to its three-fold symmetric lumen in cross-section (Fig. 3A,B). The lumen assumes a narrow three-pronged stellate shape when the radial muscles are relaxed and expands to an approximately triangular form when the radial muscles contract^41,42^. Finally, prominent circular muscles lie roughly halfway between the center and the edge of the proboscis, thereby forming a circular band defined by the three tips of the lumen^13,22^. This compartmentalized structure may facilitate independent movement of the proboscis in the anterior and posterior directions and suggests that it is associated with fluid ingestion (Fig. 3 and Supplementary Fig. S2)^43,44^.

Compared with the well-defined proboscis of the fluid-feeding species, the feeding organ of the macrophagous leech *Barbronia* sp. shows stark differences in structure. *Barbronia* sp. does not have a proboscis, instead, it has an integrated esophageal structure connected to buccal cavity with a band of circular muscles that circumscribes a tri-radiate and spacious lumen (Fig. 3D and Supplementary Fig. S2B)^45^. Intriguingly, the circular muscle of the *Alboglossiphonia* sp. proboscis is partially distributed, and the tri-radiate tips of its lumen extend further radially towards the outer band of longitudinal muscles, causing spacious (Fig. 3C,D). We speculate that morphological difference of the esophageal structure in macrophagous species compared with fluid-ingesting species is an evidence of speciation and evolutionary adaptation (Fig. 3 and Supplementary Fig. S2)^46,47^.

**Figure 4:**
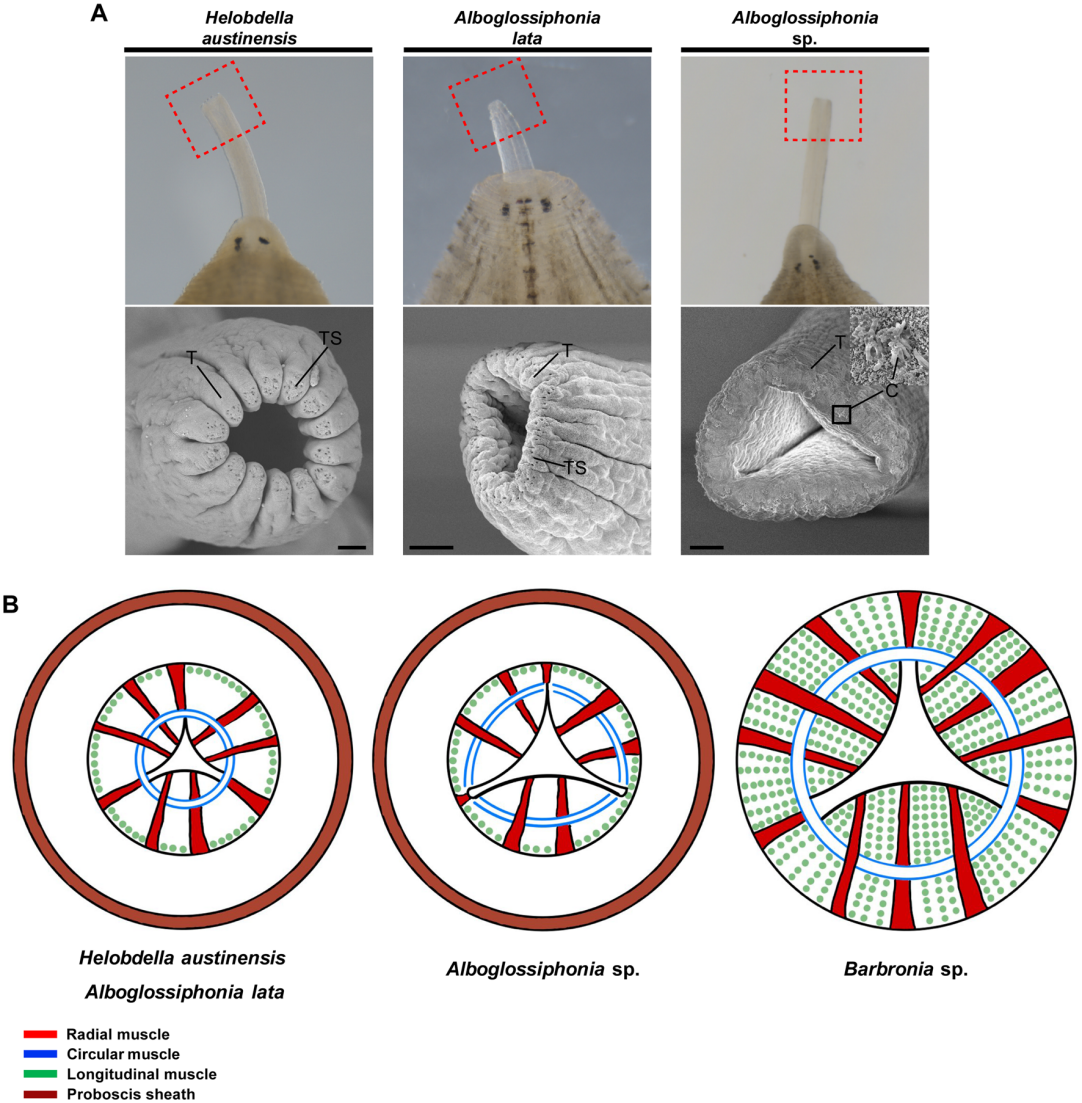
Apical structure of proboscis in glossiphoniid leeches and simplified schematics of muscle structure organization. (**A**) SEM images of proboscis tips (red dotted square) show that the tips of the proboscises of fluid-sucking species are contracted, whereas the tip of the proboscis of *Alboglossiphonia* sp. is broad and uncontracted. Numerous cilia bundles (black square) are visible at the tip of the proboscis in *Alboglossiphonia* sp. *T* tip of proboscis; *TS* secretion pore of proboscis tip; *C* cilia. Scale bars 20 μm. (**B**) Schematics of comparative muscle structure organization in leech feeding organs. Fluid ingestion leeches have compartmentalized muscle layers, with a distinct ring of circular muscles surrounding the proboscis cavity and radial muscles extending from inner to the outer region of the proboscis. In contrast, *Alboglossiphonia* sp. has three sets of separate circular muscles, radial muscles, and an expanded lumen within the proboscis. The esophagus of *Barbronia* sp. is surrounded by circular muscles with radial muscles extending throughout the body, well-developed inner radial muscles, and a lumen that expands to the circular muscle layer.

To delineate the muscular arrangement of the proboscis, we carried out molecular analyses to assess the differences in the expression of a common muscle-patterning gene and molecular markers of muscle anatomy among four leech species. Previous studies have reported on the muscular differentiation of lophotrochozoan animals. In *Platynereis dumerillii*, visceral muscles in the foregut consist of striated and smooth muscles. Within this region, troponin T proteins and myosin heavy chain (MHC) genes are involved in muscle cells and are known as annelid foregut striated muscle markers^24^. Instead of visualizing the specific *st-mhc* ortholog in each species, we tried to express the striated muscle in the esophagus in each species based on the similarity between the orthologs of *Hirudo nipponia* (Hirudinidae) and *Helobdella austinensis* (Glossiphoniidae), which are systematically distant. The two sequences showed a high level of similarity (81% identity at the nucleotide level and 93% identity at the amino acid level), suggesting that the esophageal muscle layer could be indirectly visualized using *st-mhc* orthologs of two species (Supplementary Fig. S3). In addition, nerves are distributed throughout the foregut region, and it can be assumed that muscle movement, which is controlled by the innervation pattern and the detailed arrangement of muscles in the foregut, can be confirmed by co-visualizing the nerve and muscle fibers^26^. Various muscle markers, *st-mhc* transcript, and the innervation marker acetylated tubulin show detailed intra-structural and nerve distribution according to muscle fiber in each species. Within 4 species, we confirmed that the nerve fibers are distributed along the arrangement of muscles in the esophagus (Fig. 3). Innervation of the muscle suggests that the muscles in the esophagus are regulated by neuronal stimulation, and the spatial expression of *st-mhc* orthologs reveal a potential conservation of foregut muscle components in different leech species (Fig. 3 and Supplementary Fig. S4). Fluid-ingesting leeches have a distinct longitudinal, circular, and radial muscle arrangement, and the lumen extends to the circular muscle layer (Fig. 3A,B, and Supplementary Fig. S4). The configuration of muscles in the leech proboscis exhibits structural similarity to that of vertebrate iris muscles consisting of a circular sphincter and radial muscle^13,48^ In the case of *Alboglossiphonia* sp., the circular muscle layer is partial with a spacious lumen that extends to the longitudinal muscle layer, much more so than in *H. austinensis* and *A. lata* which present distinct muscle layers (Fig. 3A–C). Due to these structural differences, the tip of the proboscis of fluid-sucking leeches shows condensed apical structure, while *Alboglossiphonia* sp. exhibits an incondensable cylindrical tip with an expanded proboscis pore (Fig. 4A). These features are likely related to the limited ability to condense the proboscis tip given the partial distribution of circular muscle, suggesting possible macrophagy via loose internal space construction (Figs. 3C,D, 4B). Also, numerous cilia bundles are clearly visible at the tip of the proboscis of *Alboglossiphoia* sp. (Fig. 4A). It is assumed that the cilia bundles are sensory cilia (sensilla) related to the recognition of prey and the proboscis is used for macrophagy^49,50^.

These results provide the first evidence suggesting that muscular organization, including differences in muscle-type composition within the proboscis, may facilitate macrophagous feeding behavior in freshwater leeches. *Barbronia* sp. is a macrophagous leech with a radial musculature in its esophagus that extends outwardly beyond the longitudinal musculature, as seen in macrophagous oligochaetes^51^, without forming a proboscis organ (Fig. 3D, Supplementary Figs. S2B and S4). This feeding organ is not an isolated proboscis, and cannot elongate or penetrate the prey, resulting in altered feeding behavior. Esophageal intramuscular complexes generate a strong suction force, resulting in a unique ingestion behavior such as swallowing of organic matter or popping of soft parts (Supplementary Movie 4). Esophageal peristalsis in vertebrates results from a complex interaction between circular and longitudinal muscles, and pushes the food toward the stomach^52^. In *Barbronia* sp., the muscular arrangement of the feeding organ is similar to that of vertebrates, suggesting that macrophagy occurs via peristalsis of longitudinal and circular muscles in the esophagus (Fig. 3D and Supplementary Fig. S2C). In addition, it is speculated that the dense longitudinal muscles located outside the esophagus may be related to the locomotion of *Barbronia* sp. (Figs. 3D and 4B). In summary, *Alboglossiphonia* sp. exhibits and alternative distribution of circular musculature, along with an expanded luminal space, designed in combination for a feeding behavior that is an intermediate between fluid-sucking and macrophagous leeches. Furthermore, this structural organization is hypothesized to facilitate a pattern of ingestion behavior similar to that of *Barbronia* sp.

### Conserved fluid ingestion behavior and compartmentalized foregut musculature in glossiphoniid juveniles

**Figure 5:**
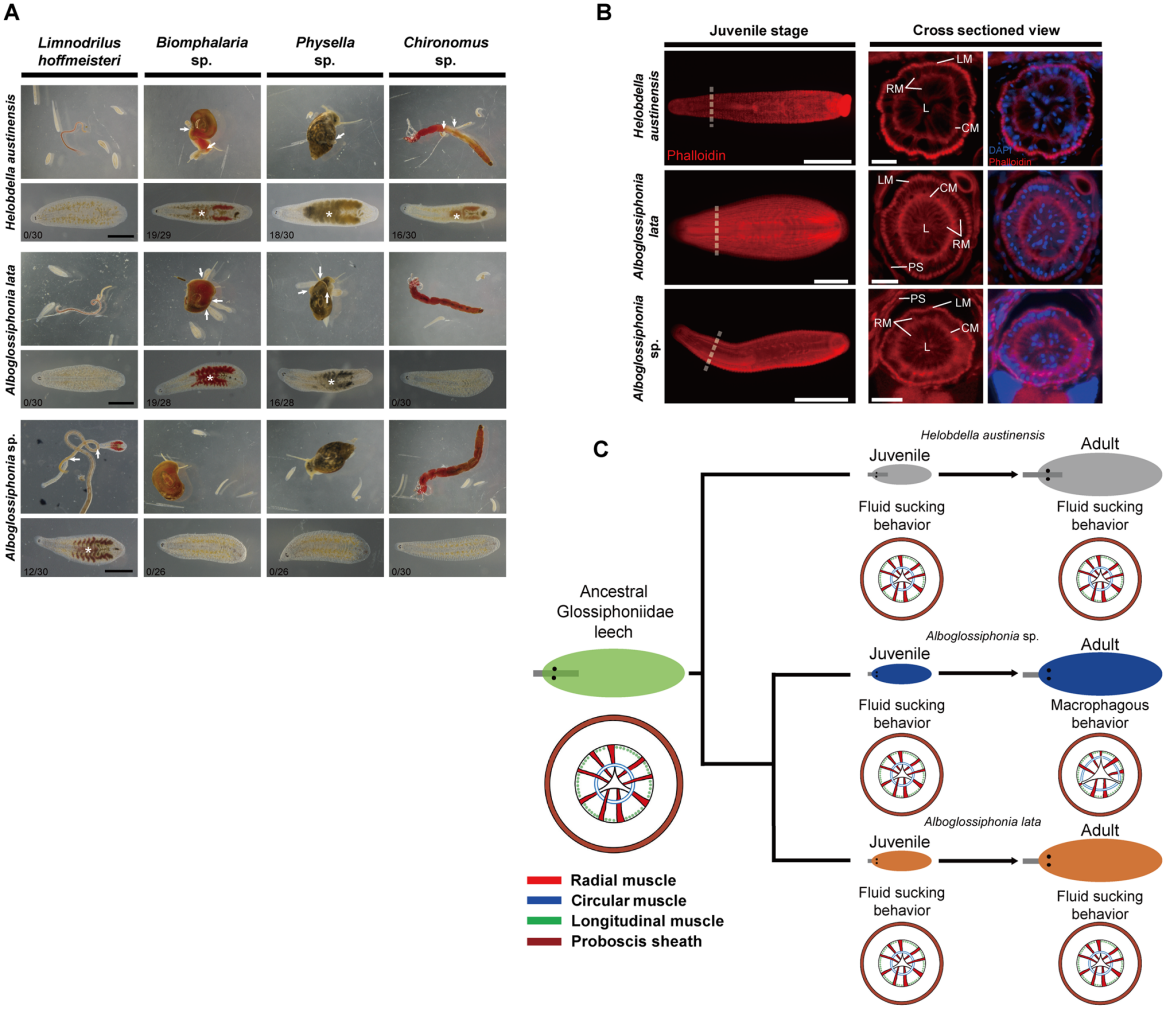
Juveniles of *Alboglossiphonia* sp. show fluid-sucking behavior and well-developed muscular structures. (**A**) Selective prey preferences among the juvenile stages of leeches. Four different prey organisms were introduced to the juvenile stages of three leech species. White arrows show the locations at which leeches fed on different species of prey. The juvenile stage of *Alboglossiphonia* sp. has the same ingestion behavior as *H. austinensis* and *A. lata*. Asterisks indicate contents in the intestine 48 h after the start of test. The numbers on the bottom left indicate the number of juveniles with filled guts compared to the total number. See Supplementary Movie 5 for ingestion behavior of *Alboglossiphonia* sp. Scale bars 500 μm. (**B**) A comparison of proboscis musculature in the juvenile stage indicates that three species have well-developed and partitioned radial muscles, as well as circular muscles in the proboscis. F-actin (red, phalloidin) and nuclei (blue, DAPI) were stained to confirm the morphology and muscle arrangement of the proboscis within juvenile leeches. White dashed lines in the first column indicate the sectioned region. CM, circular muscle; L, lumen; LM, longitudinal muscle; PC, proboscis cavity; PS, proboscis sheath; RM, radial muscle. Left column scale bars 500 μm; middle columns scale bars 20 μm. (**C**) Schematic of divergent proboscis structure according to prey preference in glossiphoniid leeches. The results of this study show that juveniles have conserved ingestion behavior and proboscis muscular structure. From a putative ancestral mechanism of feeding in glossiphoniid leeches, differences in prey preference may have influenced speciation events, and with them, changes in the morphology associated with feeding behavior. Thus far, observations suggest a conserved pattern in fluid-sucking ingestion among glossiphoniid juveniles, and at least one case of divergence to macrophagous feeding in the adult form of *Alboglossiphonia* sp.

Glossiphoniid leeches bearing a cocoon with a thin membrane have the embryos attached to the abdomen until they grow to a sufficient size. After receiving parental care, the individual leeches exhibit a parasitic life and use their developed proboscis to ingest body fluids^3,17,53–55^. However, the ingestion behavior of *Alboglossiphonia* sp. larvae is unknown apart from leeches belonging to glossiphoniid already known to be fluid-ingesting leeches. To investigate the ingestion behavior of *Alboglossiphonia* sp., we first analyzed the feeding behavior patterns of different leech species during their juvenile stages (Fig. 5A). *Alboglossiphonia* sp. adults do exhibit macrophagous feeding behavior. However, the juveniles of this species exhibit fluid-sucking behavior similar to *A. lata* and *H. austinensis* (Fig. 5A and Supplementary Movie 5). The differences in behavioral patterns are thought to be due to variation in the proboscis structure within the foregut. Thus, we conducted immunofluorescence staining to analyze the proboscis structures in juvenile stages of three species. Our analyses of fluorescent muscle markers revealed the presence of a well-developed independent proboscis in the foregut, even during the juvenile stage of food intake (Fig. 5B). Cross-sectional analyses of the juvenile proboscis showed a well-partitioned musculature in all three species, although *Alboglossiphonia* sp. showed differences between its juvenile and adult form. The arrangement of circular muscle in the adult proboscis was observed to be comparatively less structured than the other leech species, while the circular muscle layer in the juvenile stage exhibited a well-defined partition, similar to that of *H. austinensis* and *A. lata* (Figs. 3A–C, and 5B). These results indicate that *Alboglossiphonia* sp. manifests fluid-sucking behavior using well-developed muscles in the juvenile stage. Subsequently, *Alboglossiphonia* sp. undergoes gradual changes in the structural arrangement of muscles in the proboscis along with the ingestion pattern shifting to macrophagy. These findings explain the presence of an intermediate proboscis structure in *Alboglossiphonia* sp. compared with fluid-sucking and macrophagous structure seen in other leeches. Within glossiphoniid leeches, specific food preferences vary widely across species. For example, the Amazon leech *Haementeria ghilianii* is a large rhynchobdellid species adapted to feeding on mammalian blood^13,21^, *Helobdella stagnalis* consumes diverse foods including oligochaetes, molluscs, isopods, and chironomids, whereas *Glossiphonia complanata* is known as a specialist leech that preferentially feeds on molluscs^13,20^. Similarly, *A. lata* and *Alboglossiphonia* sp., which belong to the same genus, have different food niches in the same habitat (Figs. 2C and 5A). These diverse food preferences suggest that ancestral glossiphoniid leeches may have ingested a wide variety of different prey. Subsequent divergence may have arisen from the differences in morphological development that were associated with preferences for specific prey items. Furthermore, ingestion of selective prey may alter the structure of the feeding organ, and accordingly, result in differences in feeding behavior^56^. As representative examples, *H. austinensis* and *A. lata* show similar feeding behavior in the larval and adult stages, and the proboscis exhibits similar muscle structure. However, in the case of *Alboglossiphonia* sp., the larval stages ingest fluids with their proboscis, while the adults show macrophagous behavior, attributable to the differences in the arrangement of muscle layers. Therefore, within Glossiphoniidae, it appears that juvenile structural morphology facilitates ingestion of body fluids by two of the leech species we investigated, while the particular proboscis structure and feeding behavior in juvenile of *Alboglossiphonia* sp. may persist as vestiges of the ancestral, or most common, pattern observed among glossiphoniid leeches^57,58^ (Fig. 5C).

In conclusion, the results of this investigation suggest that there is an observable correlation between the internal morphological structure of the proboscis and the ingestion behavior of leech annelids. The organization of tissue and musculature in the proboscis of macrophagous leeches enables ingestion of whole organisms, unlike fluid-ingestion mechanisms. *Alboglossiphonia* sp. exhibits an esophageal structure intermediate between macrophagous and fluid-feeding leeches which manifests similar fluid intake behavior during the juvenile stage as other proboscis leeches. This behavioral pattern suggests that the feeding behavior of leeches is not intrinsic and may change depending on the development of feeding organ structure. Also, similar food preferences reveal structural and behavioral convergence among other species, despite species diversity. Genetic, morphological and behavioral differences between juvenile and adult stages of *Alboglossiphonia* sp. suggest that their adult feeding biology has diverged from ancestral glossiphoniid leeches, while retaining developmental vestiges of the typical juvenile feeding morphology currently observed across Glossiphoniidae.

## Methods

### Animal materials

Adult *Alboglossiphonia lata*, *Alboglossiphonia* sp., and *Barbronia* sp. specimens were collected by examining submerged plants, leaves, and plastic bags in selected localities of the Bangjook reservoir in Cheongju, Chungcheongbuk-do (South Korea). Adult *Glossiphonia* sp. was collected in selected localities of the Dal stream in Goesan-gun, Chungcheongbuk-do (South Korea). Adult *Hemiclepsis* sp. was collected in selected localities of the Miho stream in Cheongju, Chungcheongbuk-do (South Korea) (Supplementary Table S2). *Helobdella austinensis* was bred in the laboratory. All adult specimens except *Glossiphonia* sp. and *Hemiclepsis* sp., which cannot be incubated in artificial conditions, were incubated in a bowl containing artificial pond water. *Glossiphonia* sp. and *Hemiclepsis* sp. were fixed with 100% EtOH until used for histological analysis. The specimens were cared for once daily by changing the pond water solution and scrubbing the bowl manually to get rid of any residual waste. They were incubated in a BOD incubator at 22 °C.

### CO1 gene cloning and sequencing

Total RNA was isolated from *Alboglossiphonia* sp. embryos using TRIzol reagent (Invitrogen, Carlsbad, CA, USA). mRNA was purified from Total RNA with Oligo (dT) primer (Promega, Madison, WI, USA), and reverse transcribed into cDNA with a SuperScript II First-Strand Synthesis System for RT-PCR (Invitrogen, Carlsbad, CA USA). Genomic DNA was extracted using QIAamp DNA Mini Kit (QIAGEN, Hilden, NW, Germany). We amplified the *A. lata* CO1 gene sequences^54^ and other leech-specific CO1 and 18S rRNA genes using universal primers^27^. We used the TaKaRa Ex Taq® kit (Takara Bio Inc., Kusatsu, Japan) according to the manufacturer's instructions (predenaturation—94 °C, 5 min; denaturation—94 °C, 30 s; annealing—variable, 30 s; extension—72 °C, 1 min for COI or 1 min 30 s for 18 s rRNA sequence fragments; post extension—72 °C, 5 min).

### Phylogenetic analysis

Three partial nucleotide sequences of the mitochondrial cytochrome c oxidase subunit 1 (CO1) (*Alboglossiphonia* sp., *A. lata*, and *Barbronia* sp., about 700 bp), and four partial sequences of 18S ribosomal RNA (18S rRNA, about 1.8 kb) from the same three species plus *Helobdella austinensis* were obtained by PCR amplification. Additional sequences of both genes were obtained from the GenBank, and two alignments of 62 COI and 62 18S genes from the same group of species (see Supplementary Table S1 for GenBank accession number) were prepared using ClustalW implemented in MEGA7 software (ver. 7.0.26)^59^, and then concatenated. Phylogenetic tree hypotheses were prepared from the concatenated matrix using Maximum Likelihood (ML) and Bayesian Inference (BI). The best-fit model was searched based on the corrected Akaike Information Criterion (AICc) using IQ-TREE^60^ web-server (http://www.iqtree.org). The ML and BI analyses were conducted using RAxML-NG software (v 0.9.0)^61^ and MrBayes software (ver. 3.2.7a)^62^ under the General Time Reversible model (GTR) with a proportion of invariable sites (I) and gamma-shaped distribution rates (G4). The ML tree reconstruction was initially attempted by generating 3,000 bootstrap replicates with “autoMRE” command. The bootstrapping support values for branches were estimated under the transfer bootstrap expectation (TBE)^63^. Markov Chain Monte Carlo (MCMC) for the BI tree was run with 5,000,000 generations and the BI tree was constructed by discarding the first 25% of generations. The trees were visualized with FigTree software (ver. 1.4.4).

### Prey selection test and tracking analysis

In order to compare feeding behaviors of leeches, we conducted a survey in the laboratory environment using various food types: *Limnodrilus hoffmeisteri* (Naididae), swallowable and worm shaped; *Biomphalaria* sp. (Planorbidae) and *Physella* sp. (Physidae), unswallowable and carrying a shell; and *Chironomus* sp. (Chironomidae), unswallowable and exhibiting a worm shape. First, several individuals of each leech species were placed in a 55 mm petri-dish and ingestion patterns were observed with mixed prey species (single leech species vs. multiple prey species) and each individual prey species (single leech species vs. single prey species) to confirm exact preferences and ingestion behavior. After observation, one or two prey organisms were provided to each leech. Each experimental dish was video-recorded using a DCR-SR200 camcorder (SONY, Minato, TYO, Japan) over 8 h, or until the leeches completed feeding at room temperature. Ingestion behavior tests were performed on three biological replicates in the same condition as described above. Among the recorded videos, location analysis of ingestion behavior was conducted using one representative video for each species. To analyze the behavior of both leeches and the prey, the location of all individuals present in the petri dish was tracked every 3 min using EthoVision software (Noldus Information Technology, Wageningen, GE, Netherlands). When the predators were supplied with two species of prey, only the behavior of the prey that was ingested was tracked. However, when *Barbronia* sp. was provided with *L. hoffmeisteri* or *Chironomus* sp., the individual locations were tracked every 30 s due to their relatively rapid ingestion. Distances between leeches or preys and a reference point established on the 12 o’clock edge of the petri-dish were recorded.

### Histological analyses

To visualize differentiation of proboscis muscle structure, adult leeches were treated with relaxation buffer (4.8 mM NaCl_2_, 1.2 mM KCl, 10 mM MgCl_2_, 8% EtOH) and fixed in 4% PFA (Electron Microscopy Sciences, Hatfield, PA, USA) in 1X phosphate buffered saline (PBS) overnight at 4 °C. For H&E staining, leeches were dehydrated in EtOH series and cleared in Xylene (Central Drug House, New Delhi, DL, India) for 2 h. The leeches were embedded in paraffin (Leica, Wetzlar, HE, Germany) and stored at − 20 °C. Paraffinized samples were cut (10 µm thickness) with a RM2235 microtome (Leica, Wetzlar, HE, Germany) and stained with Mayer’s Hematoxylin (Cancer Diagnostics, Durham, NC, USA) and Eosin (Cancer Diagnostics, Durham, NC, USA). Samples were mounted on glass slides with an Organo Mount (ImmunoBioScience, Mukilteo, WA, USA) and dried overnight at room temperature. Sections were imaged with a LEICA DM6 B compound light microscope (Leica, Wetzlar, HE, Germany) and a LEICA DFC450 C camera (Leica, Wetzlar, HE, Germany). The obtained images were edited using Las X software (Leica, Wetzlar, HE, Germany) and Adobe Photoshop CS5 (Adobe, San Jose, CA, USA). The edited images were prepared as figure plates using Adobe Illustrator CS6 (Adobe, San Jose, CA, USA). To obtain cryo-sections, leeches were embedded in O.C.T. compound (VWR, Radnor, PA, USA) and rapidly frozen in liquified nitrogen. Cryo-sectioned samples (15 µm in thickness) were cut with a CM1520 cryostat (Leica, Wetzlar, HE, Germany) and stored at − 70 °C until use.

### Scanning electron microscopy of proboscis feeding organs

For scanning electron microscopy, leech specimens were treated with 16% paraformaldehyde (Electron Microscopy Sciences, Hatfield, PA, USA) or relaxation solution (4.8 mM NaCl_2_, 1.2 mM KCl, 10 mM MgCl_2_, 8% EtOH) while feeding or relaxing. After treatment, the head region containing the proboscis was cut and fixed in 4% PFA at room temperature overnight. The tissues were washed three times with PBT (1X PBS + 0.1% Tween-20) for 20 min at room temperature, and then fixed in 1% osmium tetroxide (Ted Pella Inc., Redding, CA, USA) in 1 M PBS for 1 h. Osmium tetroxide was removed by washing three times with PBT. Thereafter, the tissues were gradually dehydrated with ethanol (30%, 50%, 60%, 70%, 80%, 90%, 95%, 100% in 1X PBS) for 20 min per step. Dehydrated tissues were treated with stepwise concentrated isopentyl acetate (Alfa Aesar, Ward Hill, MA, USA) (isopentyl acetate: EtOH = 1:3, 1:1, and 3:1) for 15 min per step, and then transferred to 100% isopentyl acetate. After the solution was removed, the samples were dried for 3 days in a fume hood. Dried samples were coated with gold particles and examined with an UltraPlus field emission scanning electron microscope (Carl Zeiss, Oberkochen, BW, Germany).

### Fluorescent labeling and immunohistochemistry

Whole-mount immunostaining was performed according to previously published protocols^54^, with the following details: The cross-sections were dried and washed in PBT (0.1% Tween-20 with 1X PBS) five times. The nerve and muscle fibers were visualized after double immunostaining as follows. After washing with PBT, the sections were incubated in diluted blocking solution (1:9 = 10X Roche Western Blocking Reagent : PBT) for 2 h. Samples were incubated with primary antibodies (anti-acetylated-α-Tubulin produced in mouse, Sigma Aldrich, T-7451; or anti-cardiac TroponinT produced in rabbit, Abcam, ab115134) in diluted blocking Solution (1:500) at 4 °C for 48 h. After five consecutive washes with PBT, the sections were incubated with a secondary antibody (goat anti-mouse IgG H&L Alexa Fluor 488, Abcam, ab150113; goat anti-rabbit IgG (H + L) cross-adsorbed secondary antibody Alexa Fluor 568, Invitrogen, A11011) in diluted blocking Solution (1:1000) at 4 °C for 24 h. After checking the labeled signal, the samples were washed five times with PBT, and then stained with Texas Red™-X Phalloidin (ThermoFisher, T7471) for 1 h to visualize F-actin. After checking the labeled signal, the samples were washed five times with PBT and labeled with DAPI in PBT (1:100) at room temperature in the dark overnight. After washing with PBT five times, the samples were mounted with Fluoromount-G (SouthernBiotech, Birmingham, AL, USA). Fluorescence-stained embryos and slide samples were imaged using a LEICA DM6 B with a LEICA DFC450 C camera (Leica, Wetzlar, HE, Germany). The obtained images were edited using Las X software (Leica, Wetzlar, HE, Germany). To confirm the detailed muscle structure and innervation in the proboscis, slides co-labeled with F-actin and acetylated tubulin were imaged with a LSM 710 confocal microscope (Carl Zeiss, Oberkochen, BW, Germany). The obtained images were edited using ZEN software (L Carl Zeiss, Oberkochen, BW, Germany). The edited images were prepared as figure plates using Adobe Illustrator CS6 (Adobe, San Jose, CA, USA).

### ST-MHC gene identification, probe synthesis, and in situ hybridization

Total RNA was isolated from *H. austinensis* mixed-stage embryos and *Hirudo nipponia* head tissue using TRIzol reagent (Invitrogen, Carlsbad, CA, USA). We selected mRNA from total RNA using Oligo (dT) primer (Promega, Madison, WI, USA) and synthesized cDNA (SuperScript II First Synthesis System for RT-PCR, Invitrogen, Carlsbad, CA, USA). To isolate the *H. austinesis* striated myosin heavy chain (ST-MHC) gene, a previously published sequence^64^ was used and screened using a BLAST implemented in the whole draft-genome reference (http://genome.jgi.doe.gov/Helro1/Helro1.home.html). Two candidate genes (protein id 64,397 and 129,847) were screened, and the foregut specific *st-mhc* gene was isolated by confirming the foregut specific expression pattern (protein id: 129,847) (Supplementary Fig. S3B). In the *H. nipponia* transcriptome data, only a single striated myosin heavy chain transcript was found, which showed a high degree of similarity to the *H. austinensis* foregut specific *st-mhc* gene (nucleotide similarity: 81%, translated sequence similarity: 93%) (For nucleotide similarity, see Additional file 1: Fig S3C). The *st-mhc* specific primers were designed to amplify the consensus region of the two sequences producing similar length (product sizes about 850 nucleotides—protein id 129,847, *Hau st-mhc* forward: 5’-GCCACCAAAGGTGAAGAG-3’; *Hau st-mhc* reverse: 5’-GTCCTCAACGAGCTGCAT-3’). *H. nippoinia st-mhc* transcript (*Hni st-mhc* forward: 5’- GCCACCAAGGGCGAAGAA-3’; *Hni st-mhc* reverse: 5’- TCCTCGACCAATTGCATTTCC-3’). These amplified fragments were cloned into pGEM T vector (Promega, Madison, WI, USA). RNAprobes labeled with digoxigenin were made using the MEGAscript kit (Ambion, Austin, TX, USA) and DIG RNA Labeling Mix (Roche, Basel, Switzerland), according to the manufacturer's instructions. The synthesized RNA probes were applied to each sample at a final concentration of 2 ng/μl, and the probe labeled samples were incubated with Anti-Digoxigenin-POD Fab fragments produced in sheep (Roche, Basel, Switzerland) in diluted blocking solution (1:1000). The detail procedure of in situ hybridization was followed using previously published methods^54,65,66^. After cryosection, stored samples were dried to remove residual moisture. Dried samples were treated with 0.2 N HCl buffer to inhibit endogenous enzymes and rinsed three times with PBT. After this process, the following experiments were carried out using the same protocol as described above.

## Supplementary Information

Supplementary Information 1.

Supplementary Movie 1.

Supplementary Movie 2.

Supplementary Movie 3.

Supplementary Movie 4.

Supplementary Movie 5.

Supplementary Movie Legends.

## Data Availability

The sequences generated in this study are deposited in GenBank. GenBank accession number for phylogenetic analyses and collecting locality are presented in Supplementary Tables S1 and S2.
