# Supplementary material for: Behavioral variation according to feeding organ diversification in glossiphoniid leeches (Phylum: Annelida)
Source: Sci Rep. 2021 May 25;11:10940. doi: 10.1038/s41598-021-90421-1 (PMC8149456; doi:10.1038/s41598-021-90421-1)
Supplement: Supplementary file 1 — Supplementary Information 1. [file 41598_2021_90421_MOESM1_ESM.pdf]

Behavioral variation according to feeding organ diversification in glossiphoniid leeches (Phylum: Annelida)

Hee-Jin Kwak[1,7], Jung-Hyeuk Kim[1,2,7], Joo-Young Kim[3], Donggu Jeon[4], Doo-Hyung Lee[3], Shinja Yoo[5], Jung Kim[6], Seong-il Eyun[4], Soon Cheol Park[4*], and Sung-Jin Cho[1,*]

[1]School of Biological Sciences, College of Natural Sciences, Chungbuk National University, Cheongju, Chungbuk, 28644, Republic of Korea

[2]Wildlife Disease Response Team, National Institute of Wildlife Disease Control and Prevention, Incheon 22689, Republic of Korea

[3]Department of Life Sciences, Gachon University, Gyeonggi-do, 13120, Republic of Korea

[4]Department of Life Science, Chung-Ang University, Seoul 06974, Korea

[5]Department of Molecular and Cell Biology, University of California, 385 LSA, Berkeley, CA, 94720-3200, USA

[6]Department of Molecular and Cell Biology, University of California, 539 LSA, Berkeley, CA, 94720-3200, USA

[7]These authors contributed equally

[*]Corresponding Authors:

Sung-Jin Cho

E-mail: sjchobio@chungbuk.ac.kr

Tel: +82-43-261-2294

Soon Cheol Park

E-mail: scpark@cau.ac.kr

Tel: +82-2-820-5212

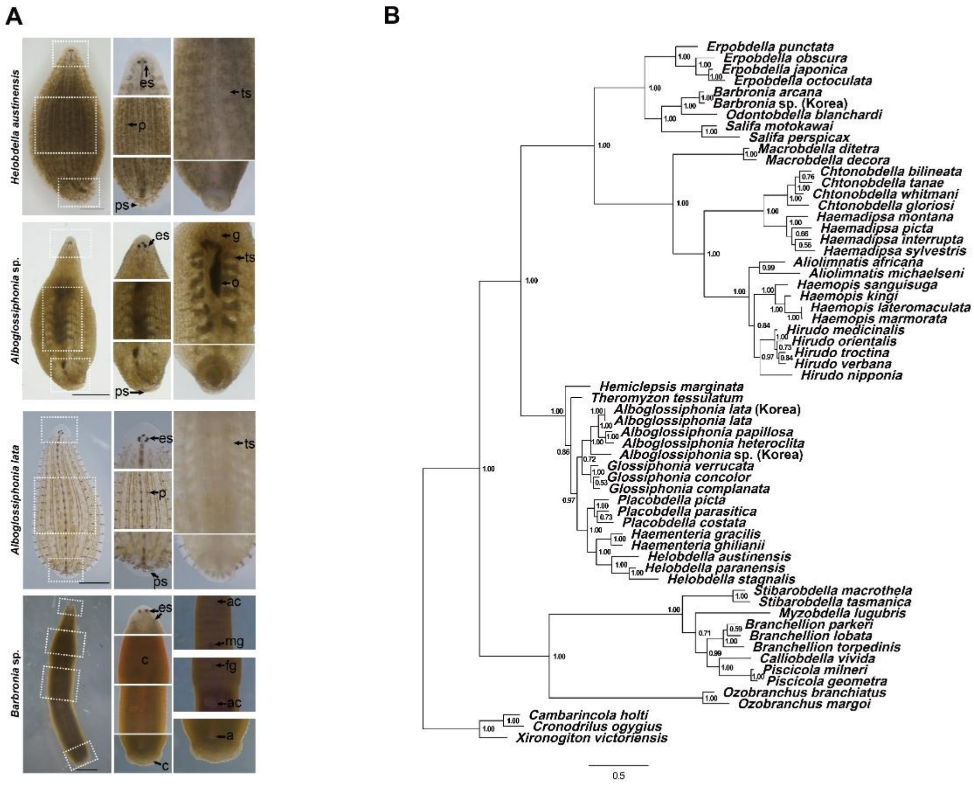

Figure S1. Morphological characters and Bayesian analysis for detail classification

(A) The external morphological comparison is based on previous studies [1-6]. White dotted boxes indicate for zoomed morphological comparison. Simplified external descriptions show the typical characteristics of each species, and the characterization of *H. austinensis* and *A. lata* is consistent with previous studies [5,7,8]. *Alboglossiphonia* sp. has characteristics of the genus *Alboglossiphonia* [2]. It has three pairs of eyes, with a typical *heteroclite* pattern, with the first pair located close together, and gonopores separated by zero annuli (data is not shown). It has six pairs of crop caeca (Fig 1C). *Barbronia* sp. shows a "worm-like" body shape similar to *B. weberi* [6,9], and typical features of genus *Barbronia* [2]. It has three pairs of cerebral eyes, with a pair of large labial eyes, followed by two pairs of eyespots. Male and female gonopores are distinguished by 4.5 annuli. The leech has two accessory copulatory pits one anterior and one posterior to the male and female gonopores, similar to *B. weberi*. a, anus; ac, accessory

copulatory pit; c, clitellum; es, eyespot; fg, female gonopore; g, gonopore; mg, male gonopore; o, ovary; p, papillae; ps, posterior sucker; ts, testisac. Scale bars 2mm.

(B) Bayesian Inference (BI) phylogenetic tree reconstructed based on the concatenated sequences of CO1 and 18S rRNA. The numbers near branches indicate Bayesian Posterior Probabilities (BPP, in probability)

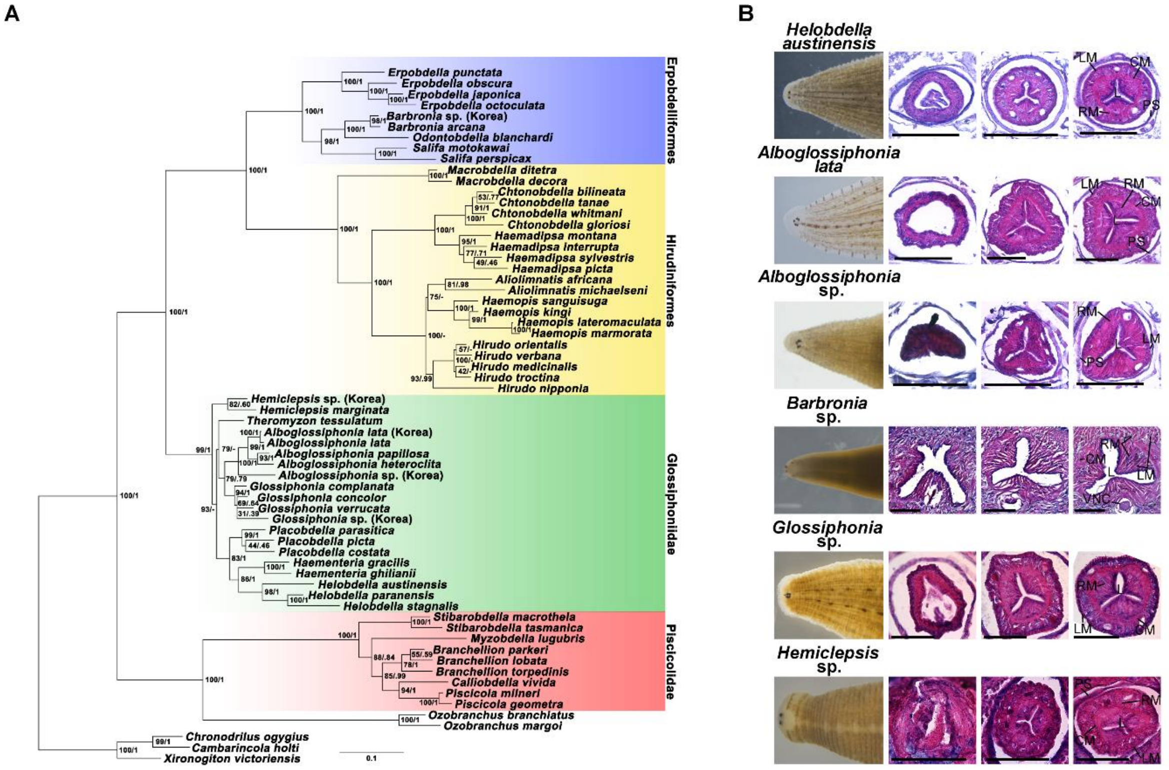

Figure S2. Phylogenetic analysis of leeches and Histological analysis in different section series

(A) A maximum likelihood (ML) phylogenetic tree reconstructed based on the concatenated sequences of CO1 and 18S rRNA including *Hemiclepsis* sp. and *Glossiphonia* sp. The numbers near branching points indicate the transfer bootstrap expectation (TBE) supports (BS, in percentage) and Bayesian Posterior Probabilities (PP, in probability) and are presented in order of "BS/PP". Dashes (-) after BS indicate PP that has not been applicable for the ML tree mainly due to the topological discrepancies between ML and Bayesian Inference (BI) trees.

(B) The distribution of compartmentalized muscle layers can be observed in continuous tissue sections in the proboscis of fluid-ingesting leeches. *Alboglossiphonia* sp. has a proboscis but does not have a clear distribution of muscle layers in histological analysis. CM, circular muscle;

LM, longitudinal muscle; PC, proboscis cavity; PS, proboscis sheath; RM, radial muscle; VNC, ventral nerve cord. Scale bars 150μm.

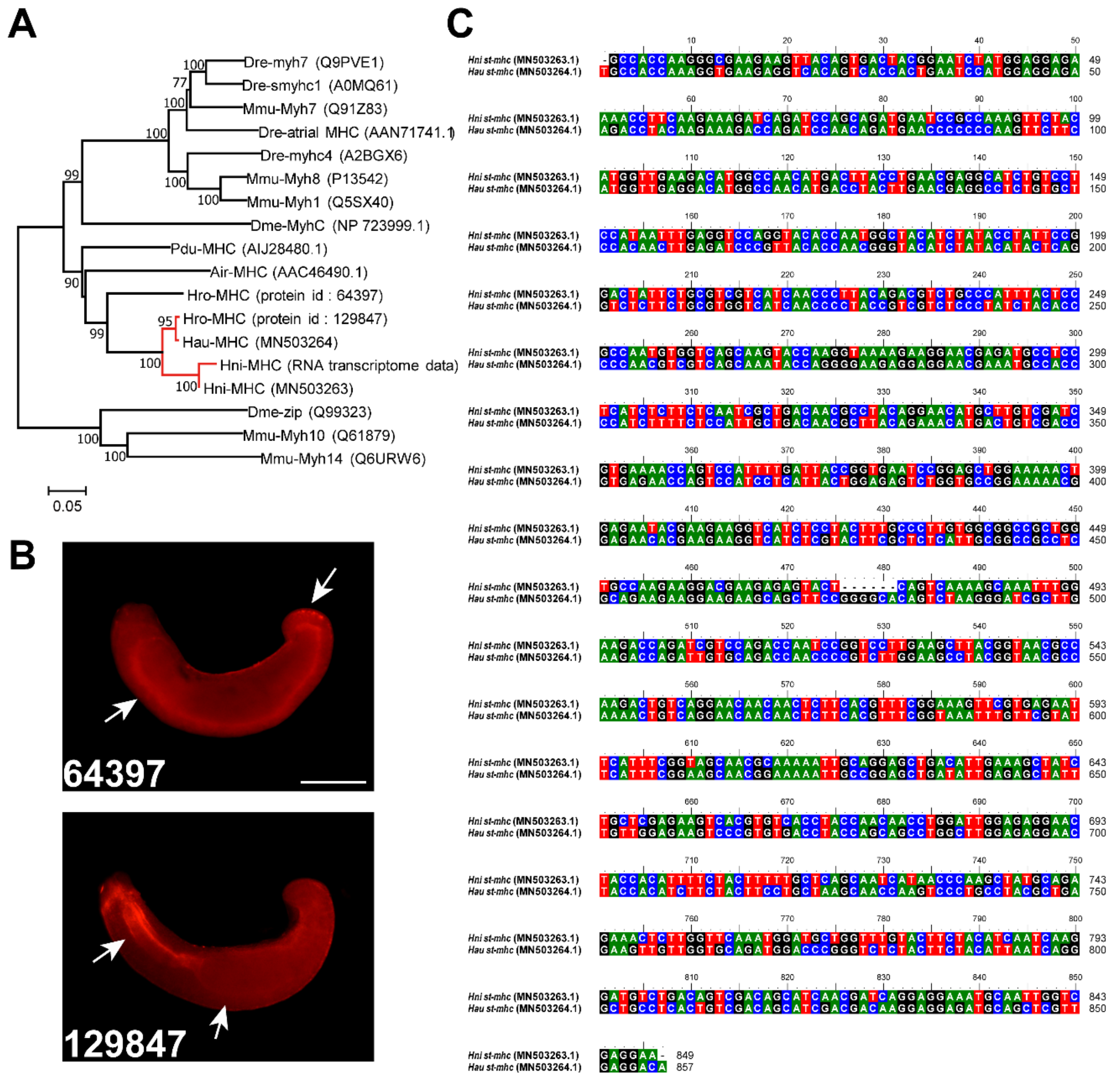

Figure S3 Foregut specific *st-mhc* identification and sequence similarity between *st-mhc* orthologs

(A) Phylogenetic analysis of Myosin Heavy chain. *H. austinensis* MHC (protein id: 129847) and *H. nipponia* MHC were clustered (red branches) in the same group, and two orthologous sequences show similarity between the two species.

(B) The two candidate *st-mhc* genes show differential expression pattern in *H. austinensis* embryo. White arrows indicate the expression regions. Protein ID 64397 was expressed in axial

longitudinal muscle within ventral region and posterior sucker muscle, whereas ID 129847 was expressed in proboscis and gut boundary. The protein ID is accessible at JGI for *Helobdella robusta* (http://genome.jgi.doe.gov/Helro1/Helro1.home.html) [10,11] Scale bar 200 μm.

(C) Alignment result using ClustalW implemented in sequence editor BioEdit (Available online: http://www.mbio.ncsu.edu/BioEdit/bioedit.html) of sequences designed for probe synthesis show sequence similarity between two *mhc* orthologs.

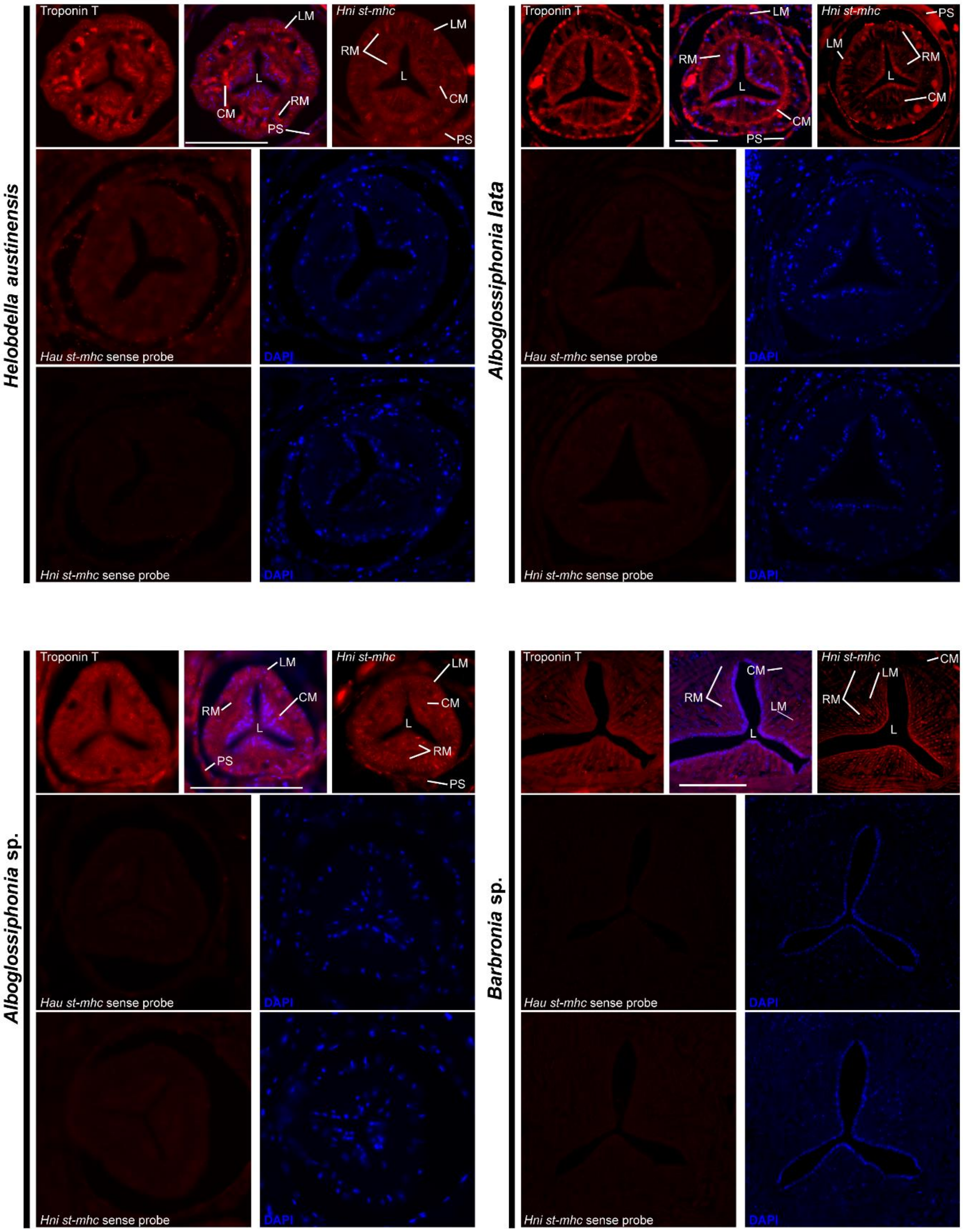

Figure S4 Expression of muscle components and spatial confirmation of the sense probes

of orthologous *st-mhc* in ingestion organs

Fluorescence expressions in esophagus indicate different muscle in fluid sucking and *Alboglossiphonia* sp. Fluorescence-labeled Troponin T proteins (top left rows) and *H. nipponia st-mhc* (*Hni st-mhc*) transcripts (top right rows) in the feeding organs of the fluid ingestion leeches were visualized in the entire muscle layers, showing typical features. In *Alboglossiphonia* sp., partial circular muscle and spacious lumen are observed. In *Barbronia* sp., a layer of circular muscles surrounding the lumen, radial muscles extending in and out of the circular muscles, and longitudinal muscles are shown. Fluorescence-labeled two orthologous *st-mhc* sense probes show no specific expression in the foregut of each leech species. DAPI staining (blue) was performed to visualize the distribution of nuclei in the entire proboscis structure. CM, circular muscle; LM, longitudinal muscle; PC, proboscis cavity; PS, proboscis sheath; RM, radial muscle. Scale bars 150μm

Species name	GenBank accession number		Species name	GenBank accession number	
	CO1	18S rRNA		CO1	18S rRNA
Xironogiton victoriensis	AF116014.1	JQ821515.1	*Piscicola milneri*	DQ414337.1	DQ414292.1
Cambarincola holti	AF116012.1	JQ821507.1	*Calliobdella vivida*	AF003260.1	AF115992.1
Cronodrilus ogygius	AF116013.1	JQ821497.1	*Macrobdella decora*	AF003271.1	AF116007.1
Ozobranchus margoi	HM590711.1	KF728217.1	*Macrobdella ditetra*	DQ097215.1	DQ097208.1
Ozobranchus branchiatus	MK386572.1	KF728214.1	*Haemadipsa sylvestris*	AF003266.1	HQ203102.1
Theromyzon tessulatum	MN393259.1	AF115980.1	*Haemadipsa interrupta*	HQ203166.1	HQ203091.1
Hemiclepsis sp. (Korea)	**MT823271**[*]	n/a	*Haemadipsa montana*	HQ203182.1	KC762968.1
Hemiclepsis marginata	MH643798.1	AF115981.1	*Haemadipsa picta*	HQ203177.1	HQ203100.1
Helobdella paranensis	AF329037.1	AF115987.1	*Chtonobdella bilineata*	HQ203159.1	KT592372.1
Helobdella stagnalis	AF116018.1	AY962416.1	*Chtonobdella tanae*	HQ203164.1	HQ203088.1
Helobdella austinensis	KC812736.1	**MT010331**[*]	*Chtonobdella whitmani*	HQ203163.1	HQ203087.1
Haementeria gracilis	AF003276.1	AF115984.1	*Chtonobdella gloriosi*	HQ203187.1	HQ203110.1
Haementeria ghilianii	AF329035.1	AF115985.1	*Aliolimnatis michaelseni*	AF116029.1	AF116010.1
Placobdella parasitica	AF003261.1	AF115990.1	*Aliolimnatis africana*	AY425451.1	AY425469.1
Placobdella picta	AF116020.1	AF115988.1	*Hirudo medicinalis*[a]	KU672396.1	AY786464.1
Placobdella costata	MF067142.1	AY962436.1	*Hirudo verbana*[a]	KU672397.1	HQ691212.1
Glossiphoia sp. (Korea)	**MT823272**[*]	n/a	*Hirudo nipponia*[a]	KC667144.1	AY425468.1
Glossiphonia complanata	AF003277.1	JQ821519.1	*Hirudo orientalis*	EF405599.1	GQ368792.1
Glossiphonia verrucata	MK479264.1	AY962432.1	*Hirudo troctina*	GQ368751.1	GQ368793.1
Glossiphonia concolor	KM095098.1	AY962431.1	*Haemopis lateromaculata*	AF116028.1	AF116009.1
Alboglossiphonia sp. (Korea)	**MN503262**[*]	**MT010328**[*]	*Haemopis marmorata*	AF003270.1	AF116008.1
*Alboglossiphonia lata (*Korea)	**MN961660**[*]	**MT010329**[*]	*Haemopis kingi*	KM611858.1	AY425466.1
Alboglossiphonia lata	MN393287.1	AY962411.1	*Haemopis sanguisuga*	AF462021.1	X91401.1
Alboglossiphonia heteroclita	AF116016.1	AF103730.1	*Erpobdella japonica*	AF116026.1	AB663648.1
Alboglossiphonia papillosa	MH286267.1	MH286273.1	*Erpobdella octoculata*	AF003274.1	AF116001.1
Myzobdella lugubris	AF003269.1	AF115994.1	*Erpobdella obscura*	JQ821638.1	JQ821523.1
Branchellion torpedinis	AF003265.1	AF115993.1	*Erpobdella punctata*	AF003275.1	HQ336380.1
Branchellion parkeri	DQ414308.1	DQ414262.1	*Barbronia* sp. (Korea)	**MN503261**[*]	**MT010330**[*]
Branchellion lobata	DQ414307.1	DQ414261.1	*Barbronia arcana*	DQ235598.1	DQ235608.1
Stibarobdella macrothela	AF116022.1	DQ414296.1	*Odontobdella blanchardi*	AB675016.1	AB663651.1
Stibarobdella tasmanica	DQ414343.1	DQ414298.1	*Salifa perspicax*	HQ336343.1	HQ336377.1
Piscicola geometra	AF003280.1	AF115995.1	*Salifa motokawai*	LC029431.1	LC029434.1

Table S1. List of leech species used for phylogenetic analysis with GenBank accession numbers.

[*] Accession numbers for newly obtained sequences in bold. n/a – not available

[a] Some partial or complete mitochondrial genomes

Species	Locality
Alboglossiphonia sp. (Korea)	South Korea: Bangjook reservoir 36°37'14.8"N 127°27'17.1"E, beneath leaf, plastic bag, and submerged plant
Alboglossiphonia lata (Korea)	South Korea: Bangjook reservoir 36°37'14.8"N 127°27'17.1"E, beneath leaf, plastic bag, and submerged plant
Barbronia sp. (Korea)	South Korea: Bangjook reservoir 36°37'14.8"N 127°27'17.1"E, beneath leaf, plastic bag, and submerged plant
Glossiphonia sp. (Korea)	South Korea: Dal stream 36°41'48"N 127°48'11.8"E, beneath stone
Hemiclepsis sp. (Korea)	South Korea: Miho stream 36°46'07.8"N 127°30'28.8"E, beneath stone

Table S2. Collecting locality for each specimen

Supplemental References.

1 Davies, R. W., Singhal, R. & Blinn, D. Erpobdella montezuma (Hirudinoidea: Erpobdellidae), a new species of freshwater leech from North America. *Canadian Journal of Zoology* **63**, 965-969 (1985).

2 Sawyer, R. T. *Leech biology and behaviour*. Vol. 2 (Clarendon Press Oxford, 1986).

3 el-Shimy, N. A. Description of a new species of Alboglossiphonia Lukin, 1976 (Hirudinea: Glossiphoniidae) from Egypt. *Zoology in the Middle East* **4**, 93-102 (1990).

4 Kutschera, U. The Golden Gate Leech Helobdella californica (Hirudinea: Glossiphoniidae): Occurrence and DNA- Based Taxonomy of a Species Restricted to San Francisco. *International review of Hydrobiology* **96**, 286-295 (2011).

5 Kutschera, U., Langguth, H., Kuo, D. H., Weisblat, D. & Shankland, M. Description of a new leech species from North America, Helobdella austinensis n. sp.(Hirudinea: Glossiphoniidae), with observations on its feeding behaviour. *Zoosystematics and Evolution* **89**, 239-246 (2013).

6 Sawyer, R. T. & Sawyer, D. R. The alien Asian leech Barbronia weberi (Blanchard, 1897)(Hirudinea: Salifidae) reported from two disjunct localities in North Carolina, United States, with observations on its biology and potential for laboratory research. *BioInvasions Records* **7**, 61-64 (2018).

7 Jiménez, B. I. M., Kwak, H.-J., Park, J.-S., Kim, J.-W. & Cho, S.-J. Developmental biology and potential use of Alboglossiphonia lata (Annelida: Hirudinea) as an "Evo-Devo" model organism. *Frontiers in zoology* **14**, 60 (2017).

8 Lai, Y.-T. & Chen, J.-H. *Leech fauna of Taiwan*. (National Taiwan University Press Taipei, 2010).

9 Pavluk, T., Pavluk, E. & Rasines, R. First record of the Asian leech Barbronia weberi (Blanchard, 1897)(Hirudinea: Arhynchobdellida: Erpobdelliformes: Salifidae) in the Iberian Peninsula. *Aquatic Invasions* **6**, S61-S64 (2011).

10 Kuo, D.-H. & Weisblat, D. A. A new molecular logic for BMP-mediated dorsoventral patterning in the leech Helobdella. *Current Biology* **21**, 1282-1288 (2011).

11 Kwak, H.-J., Park, J.-S., Medina Jiménez, B. I., Park, S. C. & Cho, S.-J. Spatiotemporal Expression of Anticoagulation Factor Antistasin in Freshwater Leeches. *International journal of molecular sciences* **20**, 3994 (2019).
