# Supplementary material for: Behavioral variation according to feeding organ diversification in glossiphoniid leeches (Phylum: Annelida)
Source: Sci Rep. 2021 May 25;11:10940. doi: 10.1038/s41598-021-90421-1 (PMC8149456; doi:10.1038/s41598-021-90421-1)
Supplement: Supplementary file 7 — Supplementary Movie Legends. [file 41598_2021_90421_MOESM7_ESM.docx]

**SUPPLEMENTARY MOVIE LEGENDS**

**Movie 1. Ingestion behavior of macrophagous leech *Alboglossiphonia* sp.**

**Movie 2. Ingestion behavior of fluid-sucking leech *H. austinensis*.**

**Movie 3. Ingestion behavior of fluid-sucking leech *A. lata*.**

**Movie 4. Ingestion behavior of macrophagous leech *Barbronia* sp.**

**Movie 5. Fluid-sucking ingestion behavior of *Alboglossiphonia* sp. larva**
